# Deep brain stimulation of the centromedian thalamic nucleus for the treatment of FIRES

**DOI:** 10.1002/epi4.12568

**Published:** 2021-12-13

**Authors:** Jasmine L. Hect, Luis D. Fernandez, William P. Welch, Taylor J. Abel

**Affiliations:** ^1^ Department of Neurological Surgery University of Pittsburgh School of Medicine Pittsburgh Pennsylvania USA; ^2^ Division of Pediatric Neurology Department of Pediatrics University of Pittsburgh School of Medicine Pittsburgh Pennsylvania USA; ^3^ Department of Bioengineering University of Pittsburgh Swanson School of Engineering Pittsburgh Pennsylvania USA

**Keywords:** critical care, drug‐resistant epilepsy, neuromodulation, pediatric epilepsy

## Abstract

Febrile infection‐related epilepsy syndrome (FIRES) is a rare, life‐threatening complication of febrile illness in previously healthy individuals followed by super‐refractory status epilepticus. Deep brain stimulation (DBS) has been demonstrated to be a promising therapy for the treatment of intractable epilepsy. Here, we present a pediatric patient with FIRES whose seizures were mitigated by acute DBS of the bilateral centromedian thalamic nucleus (CMTN). This is a previously healthy 11‐year‐old female who presented emergently with altered mental status, fever, and malaise after 1 week of lethargy, anorexia, fever, and abdominal pain. The patient began having seizures shortly after admission. After thorough workup for encephalitis and other potential etiologies, this patient was diagnosed with FIRES due to super‐refractory status epilepticus. Status epilepticus persisted despite pharmacologic management, immunotherapy, and vagus nerve stimulation. DBS of the bilateral CMTN (CM‐DBS) was pursued after 56 days of hospitalization, and she demonstrated considerable improvement in baseline mental status 30 days after DBS insertion. This report highlights application of CM‐DBS for super‐refractory status epilepticus in FIRES. This region is a diffusely connected brain region and has been shown to modulate neural networks contributing to seizure propagation and consciousness; therefore, neurostimulation is a potential therapeutic intervention for patients with super‐refractory status epilepticus.

## 1 INTRODUCTION

Febrile infection‐related epilepsy syndrome (FIRES) is a rare, life‐threatening complication of febrile illness in previously healthy individuals, who present with a nonspecific febrile illness followed by prolonged, refractory status epilepticus, with a mortality of 12% in children and 16%–27% in adults.^1^, ^2^ The consensus definition for FIRES includes the onset of refractory status epilepticus within 24 hours to 2 weeks of a febrile illness and is characteristically nonresponsive to traditional antiseizure medications, anesthetics, and immunotherapy.^1^, ^2^, ^3^ Proposed mechanisms include autoimmune etiologies and widespread activation of inflammatory pathways, although there has yet to be any reliable evidence to conclusively support either of these hypotheses.^1^ Despite the profound morbidity and mortality associated with FIRES, the etiology, pathogenesis, and optimal treatment paradigm remain poorly understood (for review^1^, ^4^).

Centromedian thalamic nucleus deep brain stimulation (CM‐DBS) is an emerging therapy for drug‐resistant multifocal or generalized epilepsy.^5^, ^6^, ^7^, ^8^, ^9^ The CMTN is a diffusely connected brain region and has been shown to modulate neural networks contributing to seizure propagation and consciousness.^10^, ^11^, ^12^ Neurostimulation of the CMTN modulates thalamocortical connectivity and is a promising therapy for the treatment of super‐refractory status epilepticus (SRSE) in the clinical setting of FIRES.^8^, ^13^, ^14^ Here, we report a pediatric patient with FIRES who was successfully treated with CM‐DBS.

## 2 CASE REPORT

A previously healthy 11‐year‐old female presented emergently with altered mental status following a 3‐day period of fever, lethargy, anorexia, headache, and nonspecific abdominal pain. She was found unresponsive to verbal and physical stimuli by parents who called EMS. Vitals at the time of presentation were T 38.2 C, HR 140, RR 24, BP 122/72, SpO_2_ 100%. The patient's first clinical seizure occurred shortly after arrival to the emergency department, with significant oxygen desaturation and full‐body stiffening lasting around 1 minute. She was treated with a loading dose of lorazepam. Evaluation including laboratory tests, head computerized tomography (CT), and lumbar puncture was unremarkable. She was started on levetiracetam, as well as empiric treatment for meningoencephalitis including vancomycin, ceftriaxone, and acyclovir.

**FIGURE 1:**
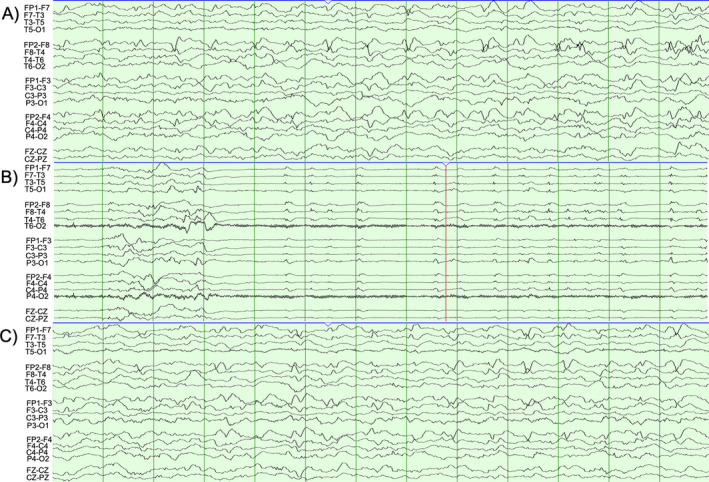
Abnormal EEG acquired on Day 1 of hospitalization, prior to sedation, demonstrating generalized background slowing and electrographic seizures captured from (A, B) right temporal and (C) bifrontal regions, without clinical correlate

Early EEG demonstrated generalized background slowing including frequent epochs of generalized rhythmic delta with superimposed fast activity and right frontotemporal epileptiform discharges, with numerous electrographic seizures arising from the right anterior temporal, right inferior frontal, or poorly lateralized over the bifrontal head regions, 30 seconds to 5 minutes in duration (Figure 1). She was transferred to the pediatric ICU where seizures persisted and increased in frequency despite escalating therapies.

**FIGURE 2:**
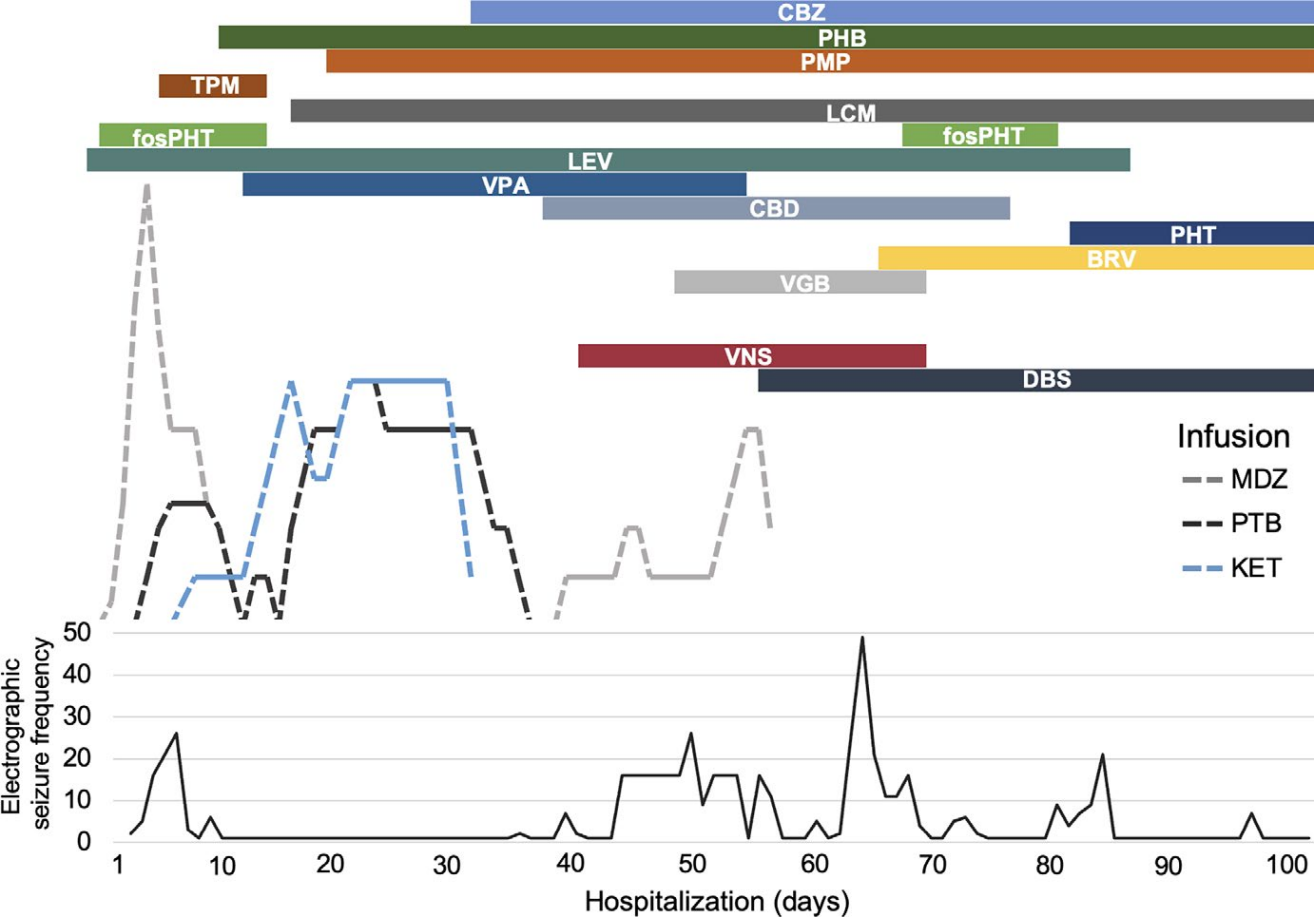
Summary of antiepileptic pharmacotherapy across hospitalization. Graphical timeline summarizing pharmacologic treatment and electrographic seizure frequency. LEV—levetiracetam =Keppra; fosPHT =fosphenytoin; PHT =phenytoin = Dilantin; TPM =topiramate = Topamax; LCM =lacosamide = Vimpat; PMP =perampanel = Fycompa; VPA =valproic acid =Depakote; PHB =phenobarbital; CBZ =clobazam = Onfi; CBD =cannabidiol = Epidiolex; VGB =vigabatrin = Sabril; BRV =brivaracetam = Briviact; MDZ =midazolam (infusion); PTB =pentobarbital (infusion); KET =ketamine (infusion). Infusion rates have been adjusted to aid visualization

Multiple antiseizure medications were introduced early in clinical course including lorazepam, levetiracetam, fosphenytoin, and lacosamide. See Figure 2 for a summary of antiseizure pharmacotherapy. On Day 2 of admission, midazolam infusion was escalated with continued electrographic seizures. The patient was sedated on Day 3 for seizure control and required a midazolam drip. On Day 4, pentobarbital infusion was initiated, with less frequent but persistent electrographic seizures arising from burst‐suppression background. Immunotherapy with IV immunoglobulin (IVIG) and high‐dose methylprednisolone was started on Day 6. Ketamine was introduced on Day 6 with resolution of electrographic seizures, and midazolam was successfully weaned. Electroclinical seizures emerged with weaning of midazolam, consisting of clonic right arm jerking, correlating with generalized periodic discharges, and electrographic seizures also re‐emerged and increased in frequency. Numerous antiseizure medications were trialed without improvement. The patient underwent five cycles of plasma exchange across 7 days, beginning on Day 21. Anakinra was started as additional immunotherapy on Day 18. Patient was weaned from anesthetics on Day 39 with subsequent increase in electrographic seizure activity characterized by predominantly right frontal multifocal epileptiform discharges. Ketogenic diet was started on Day 19, without noted improvement, and was discontinued on Day 60.

Expanded laboratory workup included negative serum and CSF autoimmune encephalitis panel, elevated CRP (1.94–2.88 mg/dL), low thyroid‐stimulating hormone (0.237) with normal T3 and free T4, normal serum and CSF studies (negative for West Nile, Bartonella, and Arbovirus antibodies), negative Lyme titers, and complement levels (C3, C4, CH50). Repeat lumbar punctures demonstrated sustained elevated opening pressure (34 cm H_2_O on Day 3 of admission, and 48 cm H_2_O on Day 13 of admission) but were otherwise unremarkable. Genetic testing was performed including comprehensive epilepsy panel (393 genes and 37 mitochondrial genes) which revealed a variant of unknown significance in GRIN2B [c.2099C>G, p.(Ala700Gly)] and carrier status for a pathogenic mutation in CLN6 [c.775G>A, p.(Gly259Ser)], neither of which were thought to be related to clinical presentation. Deletion/duplication analysis of CLN6 was eventually found to be negative.

Initial brain MRI was negative on Day 2 of admission. Repeat imaging on Day 5 showed increased perfusion in the bilateral frontal lobes and right greater than left temporal lobes but was otherwise normal. Repeat imaging on Days 15, 29, and 51 showed scattered cortical T2 hyperintensities in the bilateral internal capsule and thalami and restricted diffuse in the hippocampal tail bilaterally, as well as diffuse mild volume loss and ex vacuo ventricular enlargement, which were noted to be slightly improved on Day 57. Brain PET imaging on days 21 and 54 demonstrated broad areas of decreased metabolism with scattered focal areas of increased metabolism in the frontal lobes bilaterally. Full‐body PET did not reveal evidence of malignancy. Brain biopsy was performed on Day 57 and showed only reactive changes with no inflammatory infiltrates or evidence of infection. Immunostaining with 3F4 did not reveal evidence of prion disease.

## 3 MANAGEMENT

Neurosurgical therapy was offered for possible mitigation of this patient's SRSE. Both vagal nerve stimulation and deep brain stimulation were discussed, and the risks and benefits weighed with the patient's parents. The family opted to pursue VNS placement, although this was ultimately not successful in aborting her seizure activity, even on the highest stimulator settings (rapid cycling [58%], 2.5 output current [Magnet 2.75]). Bilateral CM‐DBS was pursued on Day 57 after discussion of risks and benefits.

**FIGURE 3:**
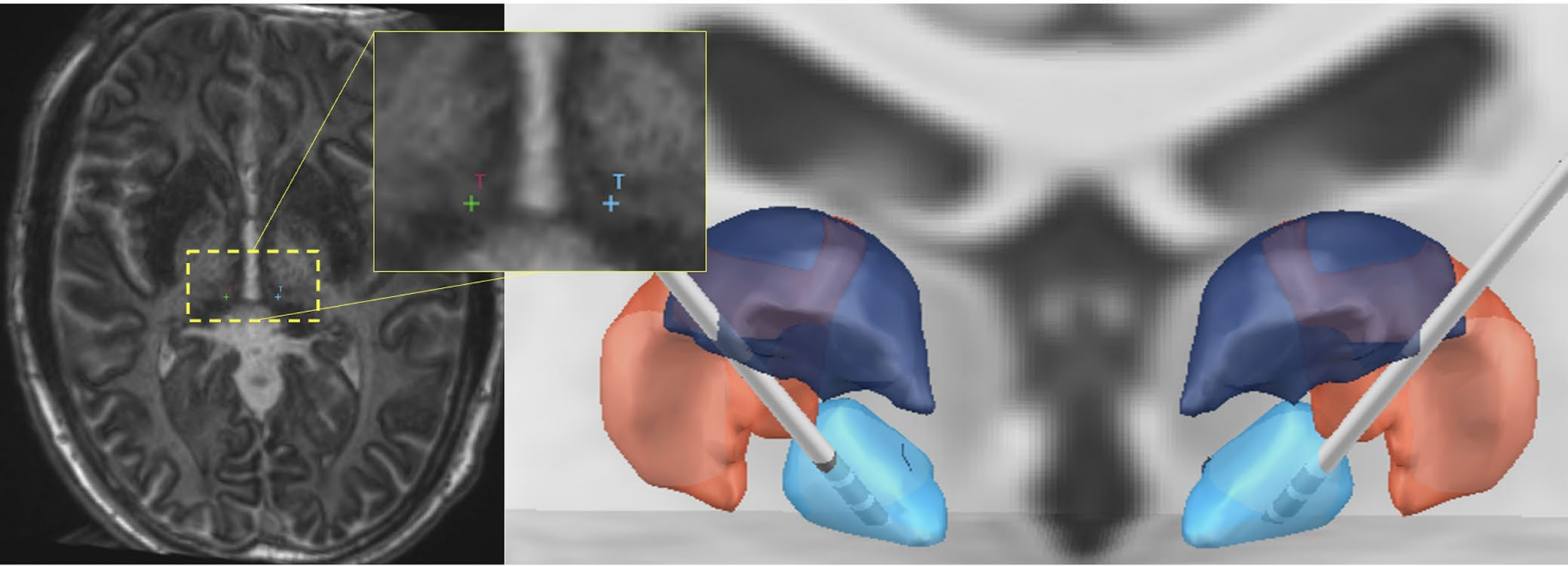
Coronal reconstruction in Lead DBS of bilateral CMTN electrode placement. Left panel: Inverse axial MP2RAGE visualized in AC‐PC orientation demonstrating good contrast of the CMTN to surrounding tissue. Preoperative electrode trajectories mapped to patient anatomy. Right panel: 3D coronal reconstruction of bilateral DBS implantation targeting the CMTN (blue), shown in relation to the poster part of the ventral posterolateral nucleus (VPLp; red) and posterior dorsal part of the ventral lateral nucleus (VLpd; purple)

Electrode trajectories were planned to the bilateral CMTN using a merged stereotactic CTA MP2RAGE MRI, MP2RAGE inversion images (Figure 3), and postcontrast MP‐RAGE MRI. Standard indirect coordinates were used and direct targeting methods using the imaging modalities described were also used, as previously reported.^8^, ^14^, ^15^, ^16^, ^17^ Trajectories were planned to avoid sulci and ventricles as well as vascular structures. Stereotactic right frontal brain biopsy was also completed at this time, targeting for which was based on the location of signal abnormality on the T2‐weighted FLAIR MRI. Intraoperative CT scan was obtained following placement of each electrode and were registered with preoperative MP2RAGE to confirm location. Boston Scientific DBS electrode leads were used and the device was initially set to amplitude 4 µV, rate 143 Hz, pulse width 90 µsec, cycling off, delivered bilaterally from the deepest contact (contact 1). Lead‐DBS software^18^ (https://www.lead‐dbs.org) was used to visualize placement in reference to thalamic nuclei defined by The Thalamus Atlas,^19^ see Figure 3.

Deep brain stimulation settings were increased on Day 63 to amplitude 5 µV, frequency 143 Hz, Pulse Width 90 µsec, with Cycling off. The patient underwent tracheostomy and percutaneous endoscopic gastrostomy tube placement on Day 69, for which her VNS and DBS were turned off. Upon attempting to turn the DBS back on to the prior settings postoperatively, the patient experienced immediate eye fluttering (left>right) with inconsistent EEG correlate. The amplitude was set to 2 µV with plans to titrate slowly back to 5 µV. The patient experienced a significant increase in number of electrographic seizures at that time (see Figure 2). DBS amplitude was increased to 2.5 µV on Day 74. Finally, DBS settings were increased adjusted on Day 85 to amplitude 3 µV and Cycling turned on (“ON time” 1 min, “OFF time” 5 min). The following day (Day 86), the patient demonstrated notable improvements in alertness and continued to improve over the next week, including ability to communicate verbally and non‐verbally (limited due to tracheostomy) and movement of extremities. The patient remained largely free from seizures through the end of her hospitalization, while she remained on continuous EEG monitoring.

Serial EEG recordings prior to discharge showed no interhemispheric voltage or frequency asymmetries, epileptiform discharges, electrographic, or electroclinical seizures. Intermittent photic stimulation was performed, using even flash frequencies between 2 and 30 flashes/second, failed to produce a driving response, and failed to activate any abnormalities. She was transferred to inpatient rehab on Day 98, where she made tremendous progress with mobility, transfers, cognition, feeding, and respiratory status. She was discharged home after 4 months of rehabilitation.

## 4 OUTCOMES AND FOLLOW‐UP

This patient presented 6 weeks after discharge from inpatient rehabilitation for increased seizure frequency, in the setting of a urinary tract infection and was discharged 2 days later. Continuous EEG during admission captured numerous electrographic seizure interictal multifocal sharp waves and numerous brief focal seizures, alternating hemispheres. Numerous brief clinical seizures consisted of alternating hemisphere rhythmic spike and wave, maximally in the temporal head regions associated with facial grimacing and drooling.

Patient underwent VNS removal 4 months after discharge from rehab for planned sEEG mapping of seizure foci at 1 month later. sEEG was pursued to aid in characterization of seizure onset zones with bilateral coverage (20 electrodes in total). Numerous daily electrographic seizures were captured arising from right frontal and left temporal regions, without clinical correlate. DBS was turned off at the time of sEEG implantation and was turned on to prior settings 48 hours later without appreciable change in seizure frequency or duration. In months following sEEG removal, the patient has not experienced clustering of her seizures and is continuing to make functional rehabilitative progress. Since sEEG mapping, she has been able to wean from perampanel and is beginning to wean from phenobarbital. She remains on a complex regimen of anticonvulsive medications overall, which her clinical team will continue to attempt to simplify, including brivaracetam, clobazam, and clonazepam for seizure clustering, phenytoin, phenobarbital, lacosamide, and intranasal midazolam for convulsive seizures.

## 5 DISCUSSION

Targeting of the CMTN for DBS is a promising rescue therapy for SRSE in FIRES, a devastating condition for which there are currently no reliable treatment options. Drug‐resistant generalized epilepsy has been shown to be responsive to neuromodulation through CM‐DBS^5^, ^6^, ^7^, ^8^, ^9^ and CM‐RNS.^15^, ^17^, ^20^, ^21^, ^22^ DBS has been utilized safely in children specifically for drug‐resistant epilepsy, including at least 40 pediatric patients (ages 4–18 years) who have received DBS treatment for epilepsy (see review^23^). There are a total of eight cases published that report the use of DBS for SRSE, in which seizure frequency decreased following implantation to the CMTN^8^, ^13^, ^24^, ^25^ or anterior thalamic nucleus (ATN).^26^, ^27^, ^28^ This suggests that DBS may be employed as a rescue therapy to reduce overall morbidity and neurologic insult related to prolonged epileptic activity and sedation. Specifically for FIRES, Sa et al (2019) reports CM‐DBS for two pediatric patients, of whom one responded positively to DBS and adjuvant immunotherapy (Anakinra).^13^ Their report demonstrated return of seizure activity when CMTN stimulation was temporarily ceased,^13^ supporting the specificity of CMTN neuromodulation in mitigating status epilepticus (vs progression of the disease from acute to chronic FIRES).^1^ Anakinra was trialed in this patient beginning on Day 18, but the patient failed to improve. Considerable work is still needed to measure the timing and stimulation parameters of CM‐DBS for the mitigation of SRSE and to understand how this therapy interacts with the course and pathogenesis of FIRES in pediatric patients.

The patient's response to DBS was promising initially, although was set back due to decreased tolerability of her prior DBS settings following DBS and VNS inactivation for OR placement of tracheostomy and PEG tube. The patient's seizures gradually decreased over the following 20 days, while DBS parameters for increased from 2 to 3µV. On Day 85, her parameters were set to 3 µV and Cycling turned on (1 min/5 min) without change to her other settings (Frequency 143 Hz, Pulse Width 90 µsec) and the patient responded with dramatic improvement in seizures and arousal the day following. The timing of response was not related to any other change in her care at that time. There is no consensus on which parameters are best for status epilepticus, and there is a wide range of reported in the literature. Most studies report seizure improvement when using frequencies of 130 Hz and 90–120 µsec pulse width.^8^, ^13^, ^24^, ^25^ Stavropoulos and colleagues most recently reported patient responsiveness to low‐frequency stimulation to the CMTN (6Hz/300 µsec pulse width).^25^ Sa and colleagues report using high‐frequency (130 Hz) stimulation to mitigate generalized seizures and later low‐frequency stimulation (6 Hz) for bifrontal focal seizures, which resulted in a transient reduction. Interestingly, Sa (2019) also describe an increase in focal seizure activity during a period of DBS inactivation. Low‐frequency stimulation has been discussed for this patient, though has not been pursued to date. The structural and functional connectivity of the CMTN supports the success of DBS in modulating seizure activity,^29^ including reciprocal connections to the striatum and cortical premotor, motor, and sensory areas, as well as direct inputs from brain stem structures (reticular formation, vestibular nucleus, solitary nucleus, and nucleus ambiguous).^30^ Stimulation of this nucleus may recruit loops of thalamocortical connectivity to aid in the termination of multifocal seizure onset seen in the setting of FIRES.^31^

Evidence of the efficacy of RNS for drug‐resistant epilepsy in pediatric patients continues to grow^15^, ^17^, ^21^, ^23^ and may offer neuromodulation approach for focal onset SRSE.^32^, ^33^ Randomized controlled trial of RNS vs DBS have not been published in the pediatric population. Recent review by Khan and colleagues (2021) found equivalent effectiveness of DBS and RNS for seizure reduction of various epilepsy etiologies.^34^ These studies suggest DBS may be more effective for multifocal, generalized SRSE and RNS for focal onset, although this has yet to be empirically tested. Future studies should investigate this systematically.

In summary, this case demonstrates the potential of bilateral CM‐DBS as a potential intervention for SRSE in FIRES. Nevertheless, the limitations of this report must be considered, including being a single case observation, complexity of disease course and unknown disease etiology. Furthermore, we are unable to separate therapeutic effects of CM‐DBS from the natural progression of FIRES in this patient to a remittent state. Further work is needed to define the pathogenesis of FIRES and trial CM‐DBS as an effective treatment for SRSE in chronic FIRES to minimize the devastating progression of this disease on cognitive and behavioral outcomes. Prospective data and clinical trials are needed to identify the optimal neurostimulation target for SRSE, given preliminary data presenting favorable outcomes for both CMTN and ATN in children.

## CONFLICTS OF INTEREST

Research reported in this publication was supported by the National Institute of General Medical Sciences of the National Institutes of Health under Award Number T32GM008208. Dr Abel reports funding from awards R21 DC019217‐01A1 and R01 DC013315‐07. Jasmine L. Hect reports funding from T32GM008208. The content is solely the responsibility of the authors and does not necessarily represent the official views of the National Institutes of Health. Dr Abel is a consultant for Monteris Medical and receives research funding through Monteris Medical for the LAANTERN Trial. We confirm that we have read the Journal's position on issues involved in ethical publication and affirm that this report is consistent with those guidelines.
